# Dok-3 deficient mice display different immune clustering and Tim-3 expression

**DOI:** 10.1186/s40001-019-0384-7

**Published:** 2019-07-27

**Authors:** Wenjiang Yan, Yijia Tian, Peng Sun, Jingjing Yang, Na Li, Yuanyuan Sun, Shuangxi Wang, Cheng Zhang

**Affiliations:** 1 The Key Laboratory of Cardiovascular Remodeling and Function Research, Chinese Ministry of Education and Chinese Ministry of Health, and The State and Shandong Province Joint Key Laboratory of Translational Cardiovascular Medicine, Qilu Hospital of Shandong University, No. 107 Wenhua Xi Road, Jinan, Shandong China; 2 Grade 2015, School of Basic Medical Sciences, Clinical Medicine(5 + 3), Shandong University, Jinan, Shandong China; 3 Key Laboratory of Infection and Immunity of Shandong Province, Jinan, China; 4 Department of Intervention Oncology, Shandong Cancer Hospital and Institute, Shandong Academy of Medical Sciences, Jinan, Shandong China

**Keywords:** Dok-3, Immune cells proportion, Tim-3

## Abstract

**Background:**

Dok-3 has been shown to play an important role in immune system. Tim-3 also has been recognized as an important immune regulator which involves in many diseases. The relationship of them is still unclear.

**Methods:**

We detected the expression of Tim-3 on spleen immune cells from Dok-3 deficient mice and control mice by flow cytometry.

**Results:**

In this article, we found that Dok-3^−/−^ mice display almost entirely different immune clustering characteristics compared with wild type 129 mice. The CD4 T cells and CD8 T cells decreased and DC cells, macrophages, MDSCs increased when the Dok-3 gene knocked-out. The Tim-3 expression on CD4 T cells, CD8 T cells, NK cells, DC cells increased when the Dok-3 gene knocked-out. The macrophages and MDSCs just display the opposite results.

**Conclusions:**

Although Dok-3^−/−^ mice display different immune clustering and Tim-3 expression, the mechanism still needs further study.

**Electronic supplementary material:**

The online version of this article (10.1186/s40001-019-0384-7) contains supplementary material, which is available to authorized users.

## Background

The Dok (downstream of kinase) family consists of seven members, with each possessing an NH2-terminal pleckstrin homology (PH), a central phosphotyrosine-binding (PTB) and a C-terminal tyrosine-rich domain [1]. Dok-1, 2 and 3 are expressed in hematopoietic cells, while the other Dok members are preferentially expressed in cells of the nervous or muscular system [2]. Dok proteins function mainly as adaptors to facilitate protein–protein interactions since they have no catalytic activity [1]. Given Dok-1 to Dok-3 are also expressed in the immune cells, they play an important role in the development of the tumor progression. Recent researches showed that they have been identified as not only tumor suppressors for lung cancer in human and mice [3] but also the depressors in the development of aggressive histiocytic sarcoma [4].

Dok-3 has been shown to play an important role in immune system. First, it can inhibit the Ca^2+^ [5] and JNK [6] activation in B cell receptor signaling. Secondly, macrophages are more sensitive to LPS-induced ERK activation with subsequent upregulation of a gene expression profile that promotes endotoxin tolerance in the absence of Dok-3 [7]. Finally, Dok-3 plays a critical and positive role in TLR3 signaling by enabling TRAF3/TBK1 complex formation and facilitating TBK1 and IFN regulatory factor 3 activation and the induction of IFN-β production [8].

T-cell immunoglobulin and mucin-domain-containing molecule 3 (Tim-3) are a membrane protein initially identified as a negative regulator of Th1 immunity [9, 10]. A strong correlation between Tim-3 expression and the immune cell function has been confirmed. Recently, Tim-3 was shown to play important roles on activated Th17 [11], Tc1 [12], macrophages/monocytes [13, 14], dendritic cells (DCs) [15] and natural killer (NK) cells [16]. However, the relationship between the Dok-3 and Tim-3 in the immune cells is still unclear. Here, we will focus on the immune cell proportion and Tim-3 expression in the Dok-3 deficient mice.

## Results

### Dok-3 deficient mice display different immune cell proportions compared with 129 mice

**Fig. 1:**
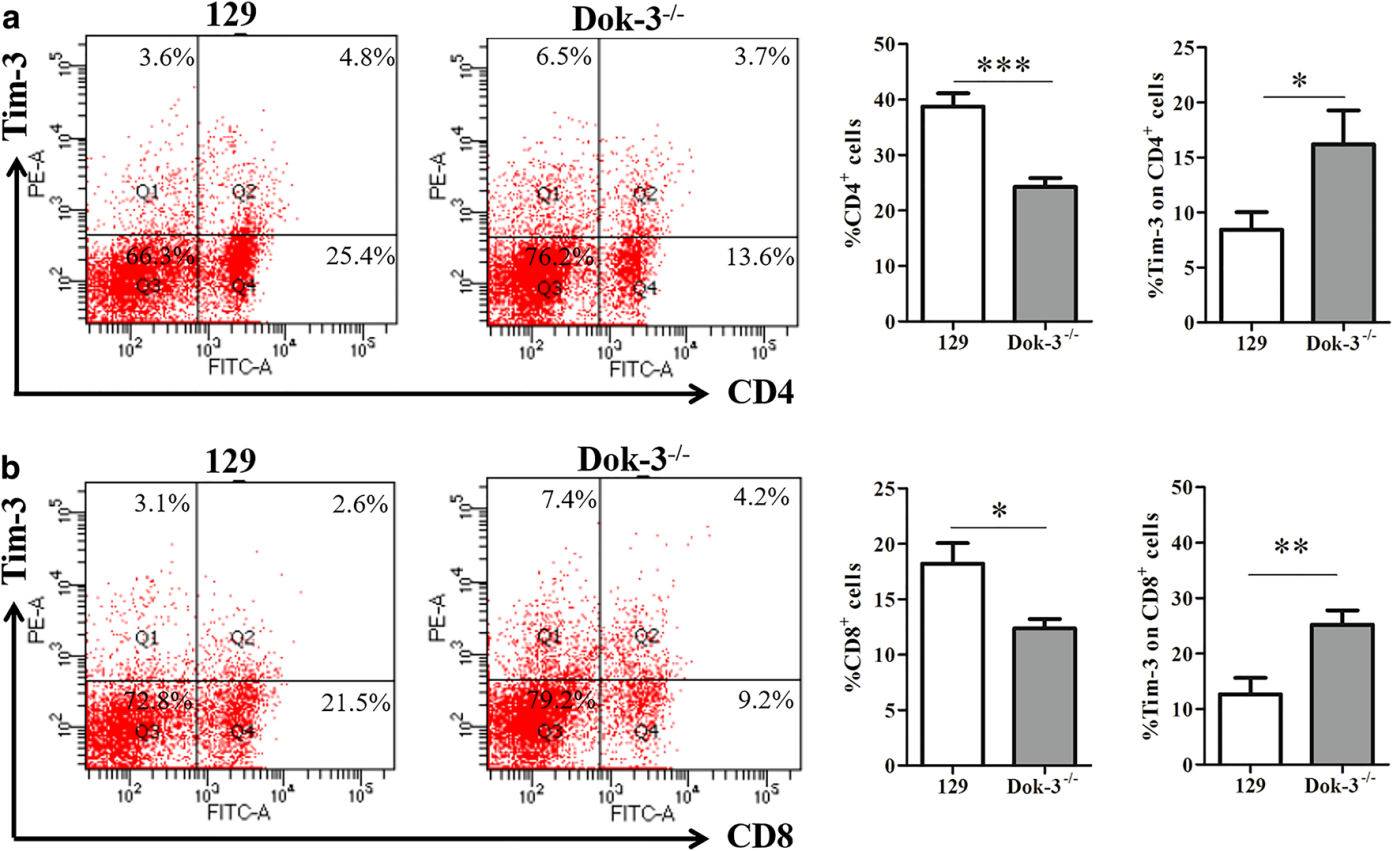
CD4^+^ T cells and CD8^+^ T cells proportion and the Tim-3 expression on these cells in two group mice. Spleen immune cells were isolated from 129 mice (*n* = 6) and Dok-3^−/−^ mice (*n* = 6). **a** Flow cytometry (FCM) analysis of Tim-3 expression on CD4^+^ T cells marked by CD3^+^CD4^+^ (left), and the statistical graph was shown (right), CD4^+^ T cells (129, 38.73 ± 2.395; Dok-3^−/−^, 24.25 ± 1.596) and Tim-3 expression (129, 8.45 ± 1.568; Dok-3^−/−^, 16.21 ± 3.064). **b** Flow cytometry (FCM) analysis of Tim-3 expression on CD8^+^ T cells marked by CD3^+^CD8^+^ (left), and the statistical graph was shown (right), CD8^+^ T cells (129, 18.20 ± 1.857; Dok-3^−/−^, 12.35 ± 0.848) and Tim-3 expression (129, 12.68 ± 2.950; Dok-3^−/−^, 25.20 ± 2.595). Both the CD4^+^ T cells and CD8^+^ T cells’ gated strategy are shown in Additional file 1: Figure S1. *p < 0.05, **p < 0.01, ***p < 0.001

**Fig. 2:**
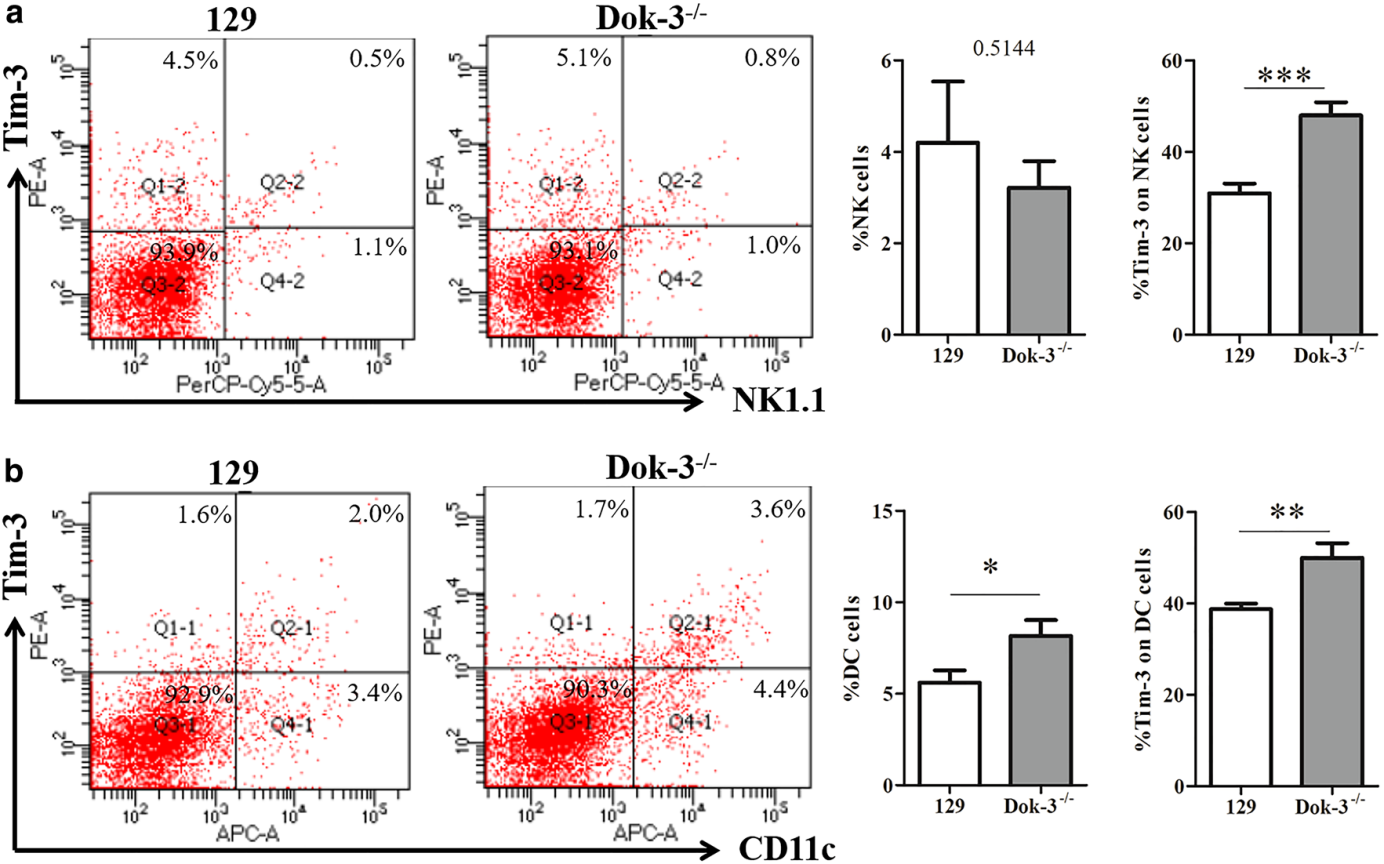
NK cells and DC cells proportion and the Tim-3 expression on these cells in two group mice. Spleen immune cells were isolated from 129 mice (*n* = 6) and Dok-3^−/−^ mice (*n* = 6). **a** Flow cytometry (FCM) analysis of Tim-3 expression on NK cells marked by CD3^−^NK1.1^+^ (left), and the statistical graph was shown (right), NK cells (129, 4.20 ± 1.335; Dok-3^−/−^, 3.22 ± 0.577) and Tim-3 expression (129, 30.97 ± 2.089; Dok-3^−/−^, 47.94 ± 2.871). **b** Flow cytometry (FCM) analysis of Tim-3 expression on DC cells marked by CD3^−^CD11c^+^ (left), and the statistical graph was shown (right), DC cells (129, 5.60 ± 0.674; Dok-3^−/−^, 8.17 ± 0.857) and Tim-3 expression (129, 38.71 ± 1.228; Dok-3^−/−^, 49.95 ± 3.191). Both the NK cells and DC cells’ gated strategy are shown in Additional file 1: Figure S1. *p < 0.05, **p < 0.01, ***p < 0.001

**Fig. 3:**
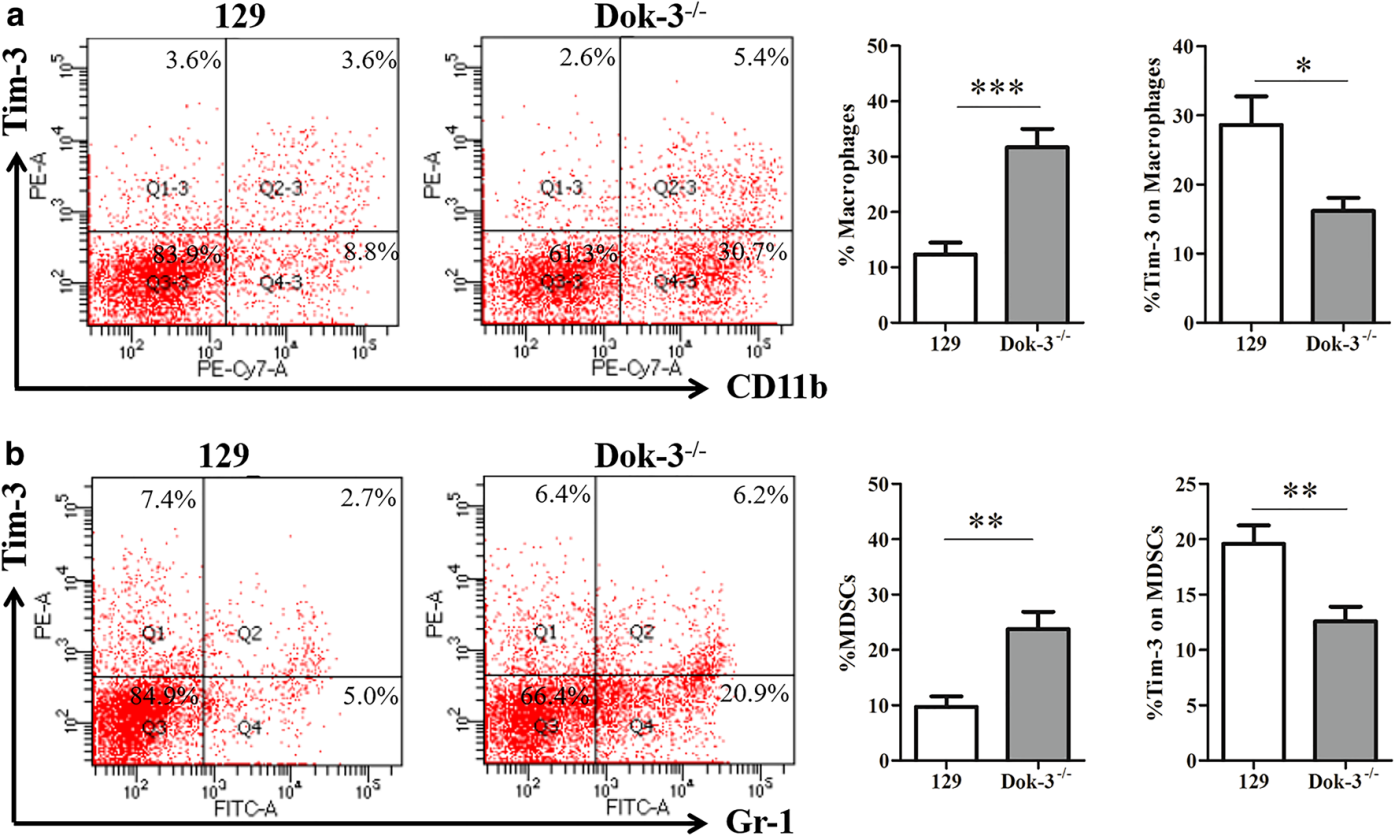
Macrophages and MDSCs proportion and the Tim-3 expression on these cells in two group mice. Spleen immune cells were isolated from 129 mice (*n* = 6) and Dok-3^−/−^ mice (*n* = 6). **a** Flow cytometry (FCM) analysis of Tim-3 expression on macrophages marked by CD3^−^CD11b^+^ (left), and the statistical graph was shown (right), macrophages (129, 12.33 ± 2.131; Dok-3^−/−^, 31.67 ± 3.336) and Tim-3 expression (129, 28.61 ± 4.120; Dok-3^−/−^, 16.18 ± 1.874). **b** Flow cytometry (FCM) analysis of Tim-3 expression on MDSCs marked by CD3^−^CD11b^+^ (left), and the statistical graph was shown (right), MDSCs (129, 9.72 ± 1.886; Dok-3^−/−^, 23.73 ± 3.150) and Tim-3 expression (129, 19.58 ± 1.661; Dok-3^−/−^, 12.58 ± 1.315). Both the macrophages and MDSCs’ gated strategy are shown in Additional file 1: Figure S1. *p < 0.05, **p < 0.01, ***p < 0.001

We analyzed the CD4 T cells, CD8 T cells, NK cells, DC cells, macrophages, MDSCs in the spleens from the two mice group. As shown in Fig. 1a, the CD4 T cells marked by CD3^+^CD4^+^ in Dok-3^−/−^ mice were much fewer than that from 129 mice (129, 38.73 ± 2.395; Dok-3^−/−^, 24.25 ± 1.596). The same tendency was found in CD8 T cells marked by CD3^+^CD8^+^ (Fig. 1b, 129, 18.20 ± 1.857; Dok-3^−/−^, 12.35 ± 0.848). In contrast, Dok-3^−/−^ mice showed much more DC cells, macrophages and MDSCs than 129 mice. As shown in Fig. 2b, DC cells marked by CD3^−^CD11c^+^ display much higher level in Dok-3^−/−^ mice compared with 129 mice (129, 5.60 ± 0.674; Dok-3^−/−^, 8.17 ± 0.857). Macrophages (CD3^−^CD11c^+^) from Dok-3^−/−^ mice were significantly higher than that from 129 mice (Fig. 3a, 129, 12.33 ± 2.131; Dok-3^−/−^, 31.67 ± 3.336). CD3^−^Gr-1^+^ was used to mark the MDSC cells (Fig. 3b). The results show that Dok-3^−/−^ mice have much more MDSCs than 129 mice (129, 9.72 ± 1.886; Dok-3^−/−^, 23.73 ± 3.150). However, the NK cells which were marked by CD3^−^NK1.1^+^ display no difference between this two group mice (Fig. 2a). In conclusion, the Dok-3^−/−^ mice display almost entirely different immune clustering characteristics compared with wild type 129 mice. The CD4 T cells and CD8 T cells decreased and DC cells, macrophages, MDSCs increased when the Dok-3 gene knocked-out.

### Dok-3 deficient mice display different Tim-3 expressions compared with 129 mice

Since Tim-3 plays an important role in the immune cells and the Dok-3^−/−^ mice display different immune cell proportions, we detected the expression of Tim-3 on these immune cells. As it is shown in Figs. 1a, b, 2a, b, the Tim-3 expression in Dok-3^−/−^ mice was much higher than that in 129 mice. This results include the CD4 cells (Fig. 1a, 129, 8.45 ± 1.568; Dok-3^−/−^, 16.21 ± 3.064), CD8 cells (Fig. 1b, 129, 12.68 ± 2.950; Dok-3^−/−^, 25.20 ± 2.595), NK cells (Fig. 2a, 129, 30.97 ± 2.089; Dok-3^−/−^, 47.94 ± 2.871), DC cells (Fig. 2b, 129, 38.71 ± 1.228; Dok-3^−/−^, 49.95 ± 3.191). Although the total NK cells number displays no difference between this two group mice, Dok-3^−/−^ mice have a much higher Tim-3 expression on NK cells than 129 mice (Fig. 2a). Contrary to the immune cells above, the macrophages (129, 28.61 ± 4.120; Dok-3^−/−^, 16.18 ± 1.874) and MDSCs (129, 19.58 ± 1.661; Dok-3^−/−^, 12.58 ± 1.315) have a much lower expression of Tim-3 in Dok-3^−/−^ mice (Fig. 3). In conclusion, the Tim-3 expression on CD4 T cells, CD8 T cells, NK cells, DC cells increased when the Dok-3 gene knocked-out. The macrophages and MDSCs just display the opposite results.

## Methods

### Mice

129 S1/SvImJ mice (129 mice) were obtained from Huafukang (Beijing, China). DOK3-deficient 129 S1/SvImJ mice were kindly provided by Mary Beth Humphrey from University of Oklahoma. The mice were housed in a pathogen-free room which could keep appropriate temperature and humidity. The mice were killed before the experiment. All animal experimental procedures were approved by the Shandong University Animal Care Committee and performed in accordance with the Animal Management Rules of the Chinese Ministry of Health.

### Spleen cell isolation and flow cytometry

Spleen immune cells were isolated from 129 mice (*n* = 6) and Dok-3^−/−^ mice (*n* = 6). Mice spleens were isolated from the body and grinded by the copper grid. The immune cells of spleen were obtained after the 10 min incubation with red blood cell lysis buffer. The cells were stained with anti-mouse Tim-3-PE (ebioscience), anti-mouse CD3-APC (ebioscience) or anti-mouse CD3-FITC (ebioscience) which was used in DC cells marking, anti-mouse CD4-FITC (ebioscience), anti-mouse CD8-FITC (ebioscience), anti-mouse CD11b-Pe-cy-7 (ebioscience), anti-mouse NK1.1-PerCP-5.5 (ebioscience), anti-mouse CD11c-APC (ebioscience), anti-mouse Gr-1-FICT (ebioscience) for 30 min. At least 10,000 cells were analyzed by a FACSAriaII. Cells were gated based on their forward and side scatter properties. The cells’ gated strategy is shown in Additional file 1: Figure S1.

### Statistical analyses

All the data were analyzed by the GraphPad Prism 5 (GraphPad Software Inc., San Diego, CA). The unpaired *t* test was used for comparison between groups. Data were reported as mean values ± SEM. Value of *p* < 0.05 was considered as significant difference.

## Discussion

In this work, we present a comprehensive analysis of the immune cells proportion difference between the 129 mice and Dok-3^−/−^ mice. We find that the CD4 T cells and CD8 T cells decreased and DC cells, macrophages, MDSCs increased when the Dok-3 gene knocked-out. NK cells display no difference between the two group mice.

Dok-3 has been demonstrated to be the key molecule in the immunity especially in the function of macrophages [5–8]. Here, we report the immune clustering alteration caused by Dok-3. The main reason why immune cells proportion changed is the proliferation, differentiation and apoptosis of these cells. But there is no evidence to prove that Dok-3 plays important role in the proliferation, differentiation and apoptosis of the immune cells. So, we still need more work to find the mechanism why Dok-3 affects the changed immune cells proportion.

Tim-3 has been recognized as an important immune regulator which expresses in both innate and adaptive immune cells. Increased evidence has shown that dysregulation of Tim-3 expression on peripheral CD4, CD8 T cells and monocytes is closely related to many diseases [11, 12, 17]. However, the roles of Dok-3 in Tim-3 expression on immune cells remain largely unknown. Here, for the first time, we demonstrated the knocked-out of Dok-3 gene which affects the Tim-3 expression on immune cells. Our data provide a previously unrecognized link between Dok-3 gene and the Tim-3 expression.

There is evidence to prove that Tim-3 is related to the apoptosis of some immune cells, especially the CD8 T cells [18]. In this study, the Dok-3^−/−^ mice display the decreased CD8 T cells and increased Tim-3 expression (Fig. 1b). Maybe the high Tim-3 expression leads to the apoptosis of CD8 T cells which is the main reason why CD8 T cells decrease.

## Conclusion

The data presented in this study suggest that Dok-3 involves in the immune cells clustering and Tim-3 expression. But the mechanism why immune cells display this proportion and difference Tim-3 expression still needs further studies.

## Additional file

**Additional file 1: Figure S1.** Gated strategy of Flow cytometry for the spleen cells.

## Data Availability

We declare that all the data and materials in this article are available.

## Abbreviations

Dok-3: downstream of kinase 3
Tim-3: T cell immunoglobulin- and mucin-domain-containing molecule-3
DC: dendritic cell
MDSC: myeloid-derived suppressor cell
