# Supplementary figures and images for: Dok-3 deficient mice display different immune clustering and Tim-3 expression
Source: Eur J Med Res. 2019 Jul 27;24:26. doi: 10.1186/s40001-019-0384-7 (PMC6660655; doi:10.1186/s40001-019-0384-7)

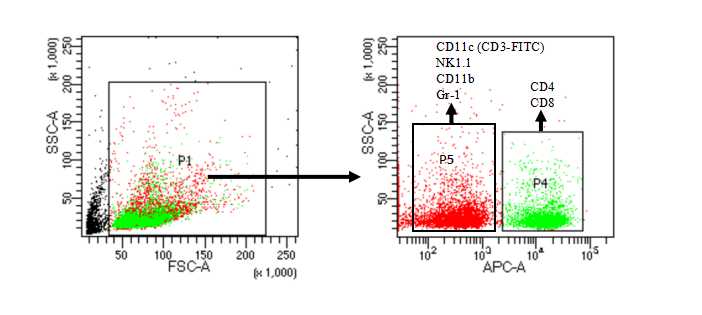

Supplement: Supplementary file 1 — Additional file 1: Figure S1. Gated strategy of Flow cytometry for the spleen cells. [file 40001_2019_384_MOESM1_ESM.jpg]
